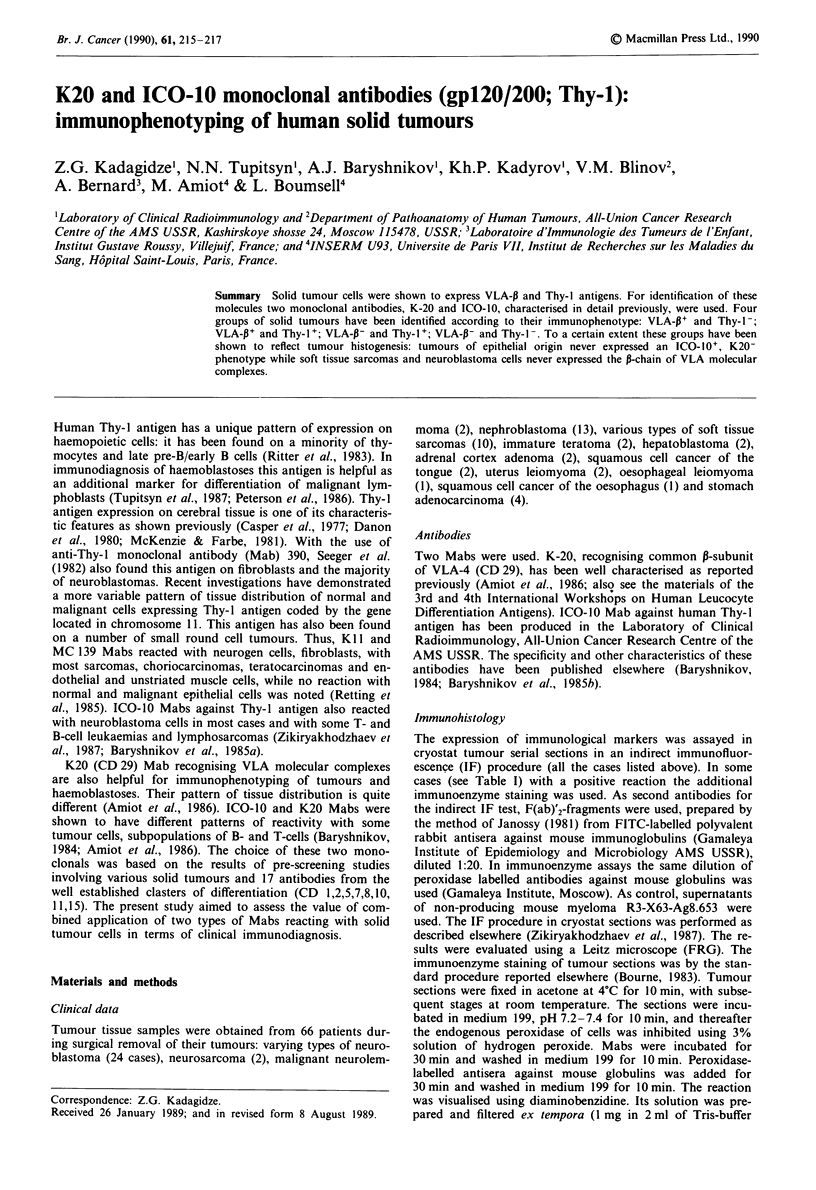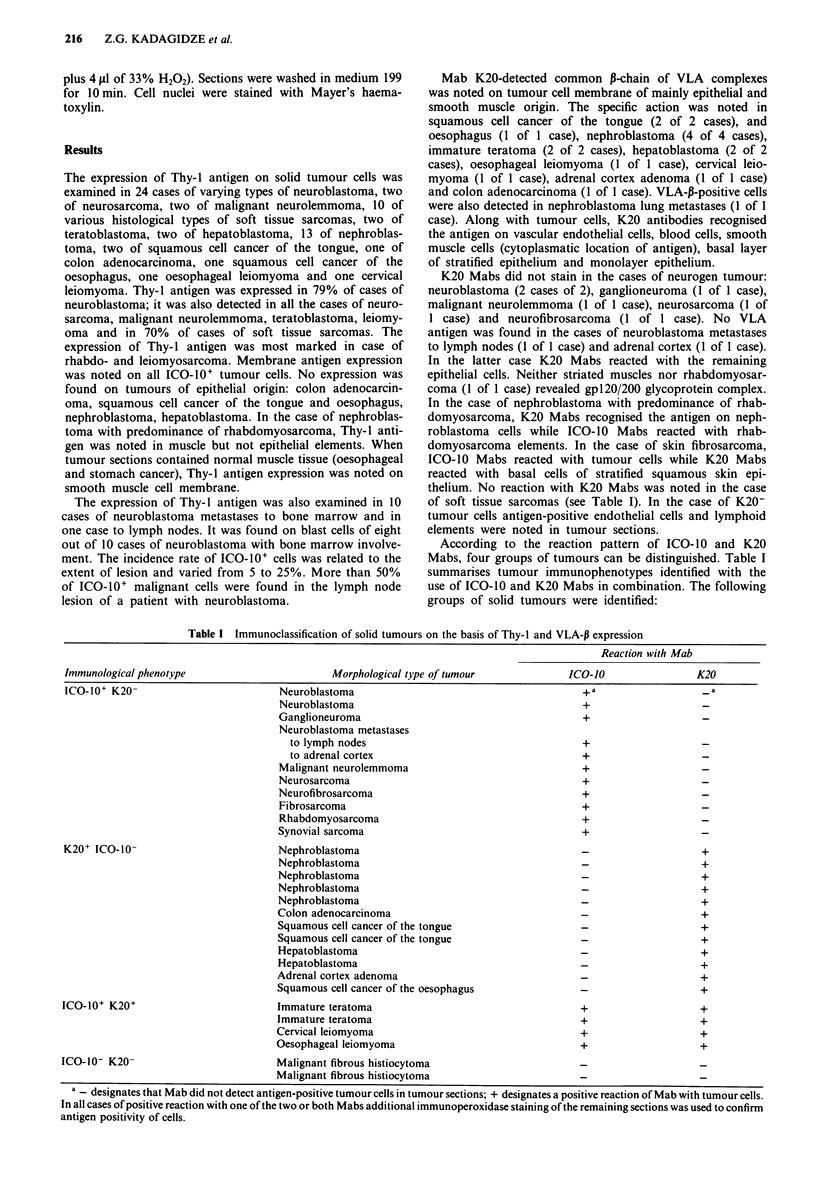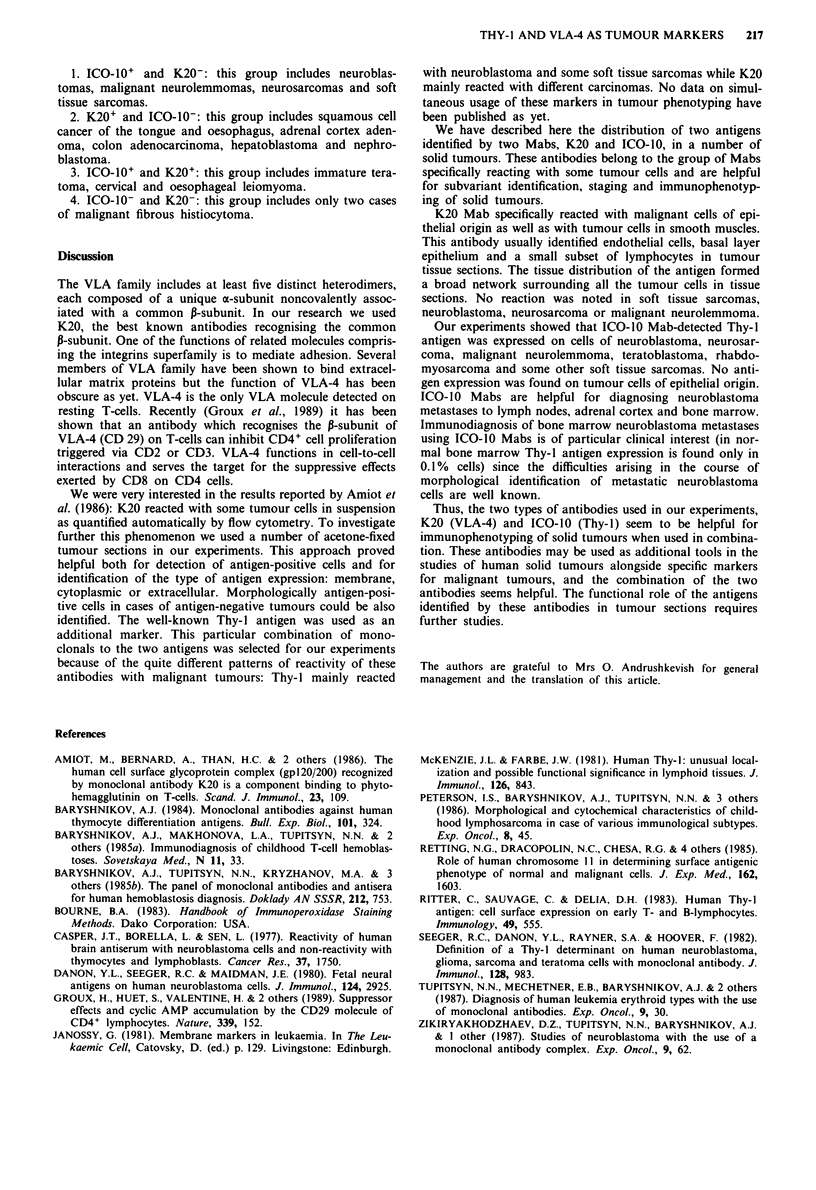# K20 and ICO-10 monoclonal antibodies (gp120/200; Thy-1): immunophenotyping of human solid tumours.

**DOI:** 10.1038/bjc.1990.39

**Published:** 1990-02

**Authors:** Z. G. Kadagidze, N. N. Tupitsyn, A. J. Baryshnikov, K. P. Kadyrov, V. M. Blinov, A. Bernard, M. Amiot, L. Boumsell

**Affiliations:** Laboratory of Clinical Radioimmunology, All-Union Cancer Research Centre of the AMS USSR, Moscow.

## Abstract

Solid tumour cells were shown to express VLA-beta and Thy-1 antigens. For identification of these molecules two monoclonal antibodies, K-20 and ICO-10, characterised in detail previously, were used. Four groups of solid tumours have been identified according to their immunophenotype: VLA-beta+ and Thy-1-; VLA-beta+ and Thy-1+; VLA-beta- and Thy-1+; VLA-beta- and Thy-1-. To a certain extent these groups have been shown to reflect tumour histogenesis: tumours of epithelial origin never expressed an ICO-10+, K20-phenotype while soft tissue sarcomas and neuroblastoma cells never expressed the beta-chain of VLA molecular complexes.


Br. J. Cancer (1990), 61, 215-217 © Macmillan Press Ltd., 1990

### K20 and ICO-10 monoclonal antibodies (gp120/200; Thy-1): immunophenotyping of human solid tumours

Z.G. Kadagidze[1], N.N. Tupitsyn[1], A.J. Baryshnikov[1], Kh.P. Kadyrov[1], V.M. Blinov[2], A. Bernard[3], M. Amiot[4] & L. Boumsell[4]

[1]*Laboratory of Clinical Radioimmunology and* [2]*Department of Pathoanatomy of Human Tumours, All-Union Cancer Research Centre of the AMS USSR, Kashirskoye shosse 24, Moscow 115478, USSR;* [3]*Laboratoire d'Immunologie des Tumeurs de l'Enfant, Institut Gustave Roussy, Villejuif, France; and* [4]*INSERM U93, Universite de Paris VII, Institut de Recherches sur les Maladies du Sang, Hôpital Saint-Louis, Paris, France.*

**Summary** Solid tumour cells were shown to express VLA-$\beta$ and Thy-1 antigens. For identification of these molecules two monoclonal antibodies, K-20 and ICO-10, characterised in detail previously, were used. Four groups of solid tumours have been identified according to their immunophenotype: VLA-$\beta^+$ and Thy-$1^-$; VLA-$\beta^+$ and Thy-$1^+$; VLA-$\beta^-$ and Thy-$1^+$; VLA-$\beta^-$ and Thy-$1^-$. To a certain extent these groups have been shown to reflect tumour histogenesis: tumours of epithelial origin never expressed an ICO-$10^+$, $K20^-$ phenotype while soft tissue sarcomas and neuroblastoma cells never expressed the $\beta$-chain of VLA molecular complexes.

Human Thy-1 antigen has a unique pattern of expression on haemopoietic cells: it has been found on a minority of thymocytes and late pre-B/early B cells (Ritter *et al.*, 1983). In immunodiagnosis of haemoblastoses this antigen is helpful as an additional marker for differentiation of malignant lymphoblasts (Tupitsyn *et al.*, 1987; Peterson *et al.*, 1986). Thy-1 antigen expression on cerebral tissue is one of its characteristic features as shown previously (Casper *et al.*, 1977; Danon *et al.*, 1980; McKenzie & Farbe, 1981). With the use of anti-Thy-1 monoclonal antibody (Mab) 390, Seeger *et al.* (1982) also found this antigen on fibroblasts and the majority of neuroblastomas. Recent investigations have demonstrated a more variable pattern of tissue distribution of normal and malignant cells expressing Thy-1 antigen coded by the gene located in chromosome 11. This antigen has also been found on a number of small round cell tumours. Thus, K11 and MC 139 Mabs reacted with neurogen cells, fibroblasts, with most sarcomas, choriocarcinomas, teratocarcinomas and endothelial and unstriated muscle cells, while no reaction with normal and malignant epithelial cells was noted (Retting *et al.*, 1985). ICO-10 Mabs against Thy-1 antigen also reacted with neuroblastoma cells in most cases and with some T- and B-cell leukaemias and lymphosarcomas (Zikiryakhodzhaev *et al.*, 1987; Baryshnikov *et al.*, 1985*a*).

K20 (CD 29) Mab recognising VLA molecular complexes are also helpful for immunophenotyping of tumours and haemoblastoses. Their pattern of tissue distribution is quite different (Amiot *et al.*, 1986). ICO-10 and K20 Mabs were shown to have different patterns of reactivity with some tumour cells, subpopulations of B- and T-cells (Baryshnikov, 1984; Amiot *et al.*, 1986). The choice of these two monoclonals was based on the results of pre-screening studies involving various solid tumours and 17 antibodies from the well established clasters of differentiation (CD 1,2,5,7,8,10, 11,15). The present study aimed to assess the value of combined application of two types of Mabs reacting with solid tumour cells in terms of clinical immunodiagnosis.

### Materials and methods

### *Clinical data*

Tumour tissue samples were obtained from 66 patients during surgical removal of their tumours: varying types of neuroblastoma (24 cases), neurosarcoma (2), malignant neurolemmoma (2), nephroblastoma (13), various types of soft tissue sarcomas (10), immature teratoma (2), hepatoblastoma (2), adrenal cortex adenoma (2), squamous cell cancer of the tongue (2), uterus leiomyoma (2), oesophageal leiomyoma (1), squamous cell cancer of the oesophagus (1) and stomach adenocarcinoma (4).

### *Antibodies*

Two Mabs were used. K-20, recognising common $\beta$-subunit of VLA-4 (CD 29), has been well characterised as reported previously (Amiot *et al.*, 1986; also see the materials of the 3rd and 4th International Workshops on Human Leucocyte Differentiation Antigens). ICO-10 Mab against human Thy-1 antigen has been produced in the Laboratory of Clinical Radioimmunology, All-Union Cancer Research Centre of the AMS USSR. The specificity and other characteristics of these antibodies have been published elsewhere (Baryshnikov, 1984; Baryshnikov *et al.*, 1985*b*).

### *Immunohistology*

The expression of immunological markers was assayed in cryostat tumour serial sections in an indirect immunofluorescence (IF) procedure (all the cases listed above). In some cases (see Table I) with a positive reaction the additional immunoenzyme staining was used. As second antibodies for the indirect IF test, $F(ab)'_2$-fragments were used, prepared by the method of Janossy (1981) from FITC-labelled polyvalent rabbit antisera against mouse immunoglobulins (Gamaleya Institute of Epidemiology and Microbiology AMS USSR), diluted 1:20. In immunoenzyme assays the same dilution of peroxidase labelled antibodies against mouse globulins was used (Gamaleya Institute, Moscow). As control, supernatants of non-producing mouse myeloma R3-X63-Ag8.653 were used. The IF procedure in cryostat sections was performed as described elsewhere (Zikiryakhodzhaev *et al.*, 1987). The results were evaluated using a Leitz microscope (FRG). The immunoenzyme staining of tumour sections was by the standard procedure reported elsewhere (Bourne, 1983). Tumour sections were fixed in acetone at 4°C for 10 min, with subsequent stages at room temperature. The sections were incubated in medium 199, pH 7.2-7.4 for 10 min, and thereafter the endogenous peroxidase of cells was inhibited using 3% solution of hydrogen peroxide. Mabs were incubated for 30 min and washed in medium 199 for 10 min. Peroxidase-labelled antisera against mouse globulins was added for 30 min and washed in medium 199 for 10 min. The reaction was visualised using diaminobenzidine. Its solution was prepared and filtered *ex tempora* (1 mg in 2 ml of Tris-buffer

Correspondence: Z.G. Kadagidze.
Received 26 January 1989; and in revised form 8 August 1989.

plus 4 μl of 33% $H_2O_2$). Sections were washed in medium 199 for 10 min. Cell nuclei were stained with Mayer's haematoxylin.

### Results

The expression of Thy-1 antigen on solid tumour cells was examined in 24 cases of varying types of neuroblastoma, two of neurosarcoma, two of malignant neurolemmoma, 10 of various histological types of soft tissue sarcomas, two of teratoblastoma, two of hepatoblastoma, 13 of nephroblastoma, two of squamous cell cancer of the tongue, one of colon adenocarcinoma, one squamous cell cancer of the oesophagus, one oesophageal leiomyoma and one cervical leiomyoma. Thy-1 antigen was expressed in 79% of cases of neuroblastoma; it was also detected in all the cases of neurosarcoma, malignant neurolemmoma, teratoblastoma, leiomyoma and in 70% of cases of soft tissue sarcomas. The expression of Thy-1 antigen was most marked in case of rhabdo- and leiomyosarcoma. Membrane antigen expression was noted on all ICO-10$^+$ tumour cells. No expression was found on tumours of epithelial origin: colon adenocarcinoma, squamous cell cancer of the tongue and oesophagus, nephroblastoma, hepatoblastoma. In the case of nephroblastoma with predominance of rhabdomyosarcoma, Thy-1 antigen was noted in muscle but not epithelial elements. When tumour sections contained normal muscle tissue (oesophageal and stomach cancer), Thy-1 antigen expression was noted on smooth muscle cell membrane.

The expression of Thy-1 antigen was also examined in 10 cases of neuroblastoma metastases to bone marrow and in one case to lymph nodes. It was found on blast cells of eight out of 10 cases of neuroblastoma with bone marrow involvement. The incidence rate of ICO-10$^+$ cells was related to the extent of lesion and varied from 5 to 25%. More than 50% of ICO-10$^+$ malignant cells were found in the lymph node lesion of a patient with neuroblastoma.

Mab K20-detected common β-chain of VLA complexes was noted on tumour cell membrane of mainly epithelial and smooth muscle origin. The specific action was noted in squamous cell cancer of the tongue (2 of 2 cases), and oesophagus (1 of 1 case), nephroblastoma (4 of 4 cases), immature teratoma (2 of 2 cases), hepatoblastoma (2 of 2 cases), oesophageal leiomyoma (1 of 1 case), cervical leiomyoma (1 of 1 case), adrenal cortex adenoma (1 of 1 case) and colon adenocarcinoma (1 of 1 case). VLA-β-positive cells were also detected in nephroblastoma lung metastases (1 of 1 case). Along with tumour cells, K20 antibodies recognised the antigen on vascular endothelial cells, blood cells, smooth muscle cells (cytoplasmatic location of antigen), basal layer of stratified epithelium and monolayer epithelium.

K20 Mabs did not stain in the cases of neurogen tumour: neuroblastoma (2 cases of 2), ganglioneuroma (1 of 1 case), malignant neurolemmoma (1 of 1 case), neurosarcoma (1 of 1 case) and neurofibrosarcoma (1 of 1 case). No VLA antigen was found in the cases of neuroblastoma metastases to lymph nodes (1 of 1 case) and adrenal cortex (1 of 1 case). In the latter case K20 Mabs reacted with the remaining epithelial cells. Neither striated muscles nor rhabdomyosarcoma (1 of 1 case) revealed gp120/200 glycoprotein complex. In the case of nephroblastoma with predominance of rhabdomyosarcoma, K20 Mabs recognised the antigen on nephroblastoma cells while ICO-10 Mabs reacted with rhabdomyosarcoma elements. In the case of skin fibrosarcoma, ICO-10 Mabs reacted with tumour cells while K20 Mabs reacted with basal cells of stratified squamous skin epithelium. No reaction with K20 Mabs was noted in the case of soft tissue sarcomas (see Table I). In the case of K20$^-$ tumour cells antigen-positive endothelial cells and lymphoid elements were noted in tumour sections.

According to the reaction pattern of ICO-10 and K20 Mabs, four groups of tumours can be distinguished. Table I summarises tumour immunophenotypes identified with the use of ICO-10 and K20 Mabs in combination. The following groups of solid tumours were identified:

**Table I** Immunoclassification of solid tumours on the basis of Thy-1 and VLA-β expression

| | | *Reaction with Mab* | |
|---|---|---|---|
| *Immunological phenotype* | *Morphological type of tumour* | *ICO-10* | *K20* |
| ICO-10$^+$ K20$^-$ | Neuroblastoma | +[a] | –[a] |
| | Neuroblastoma | + | – |
| | Ganglioneuroma | + | – |
| | Neuroblastoma metastases | | |
| | to lymph nodes | + | – |
| | to adrenal cortex | + | – |
| | Malignant neurolemmoma | + | – |
| | Neurosarcoma | + | – |
| | Neurofibrosarcoma | + | – |
| | Fibrosarcoma | + | – |
| | Rhabdomyosarcoma | + | – |
| | Synovial sarcoma | + | – |
| K20$^+$ ICO-10$^-$ | Nephroblastoma | – | + |
| | Nephroblastoma | – | + |
| | Nephroblastoma | – | + |
| | Nephroblastoma | – | + |
| | Nephroblastoma | – | + |
| | Colon adenocarcinoma | – | + |
| | Squamous cell cancer of the tongue | – | + |
| | Squamous cell cancer of the tongue | – | + |
| | Hepatoblastoma | – | + |
| | Hepatoblastoma | – | + |
| | Adrenal cortex adenoma | – | + |
| | Squamous cell cancer of the oesophagus | – | + |
| ICO-10$^+$ K20$^+$ | Immature teratoma | + | + |
| | Immature teratoma | + | + |
| | Cervical leiomyoma | + | + |
| | Oesophageal leiomyoma | + | + |
| ICO-10$^-$ K20$^-$ | Malignant fibrous histiocytoma | – | – |
| | Malignant fibrous histiocytoma | – | – |

[a] – designates that Mab did not detect antigen-positive tumour cells in tumour sections; + designates a positive reaction of Mab with tumour cells. In all cases of positive reaction with one of the two or both Mabs additional immunoperoxidase staining of the remaining sections was used to confirm antigen positivity of cells.

1. ICO-10$^+$ and K20$^-$: this group includes neuroblastomas, malignant neurolemmomas, neurosarcomas and soft tissue sarcomas.

2. K20$^+$ and ICO-10$^-$: this group includes squamous cell cancer of the tongue and oesophagus, adrenal cortex adenoma, colon adenocarcinoma, hepatoblastoma and nephroblastoma.

3. ICO-10$^+$ and K20$^+$: this group includes immature teratoma, cervical and oesophageal leiomyoma.

4. ICO-10$^-$ and K20$^-$: this group includes only two cases of malignant fibrous histiocytoma.

### Discussion

The VLA family includes at least five distinct heterodimers, each composed of a unique $\alpha$-subunit noncovalently associated with a common $\beta$-subunit. In our research we used K20, the best known antibodies recognising the common $\beta$-subunit. One of the functions of related molecules comprising the integrins superfamily is to mediate adhesion. Several members of VLA family have been shown to bind extracellular matrix proteins but the function of VLA-4 has been obscure as yet. VLA-4 is the only VLA molecule detected on resting T-cells. Recently (Groux *et al.*, 1989) it has been shown that an antibody which recognises the $\beta$-subunit of VLA-4 (CD 29) on T-cells can inhibit CD4$^+$ cell proliferation triggered via CD2 or CD3. VLA-4 functions in cell-to-cell interactions and serves the target for the suppressive effects exerted by CD8 on CD4 cells.

We were very interested in the results reported by Amiot *et al.* (1986): K20 reacted with some tumour cells in suspension as quantified automatically by flow cytometry. To investigate further this phenomenon we used a number of acetone-fixed tumour sections in our experiments. This approach proved helpful both for detection of antigen-positive cells and for identification of the type of antigen expression: membrane, cytoplasmic or extracellular. Morphologically antigen-positive cells in cases of antigen-negative tumours could be also identified. The well-known Thy-1 antigen was used as an additional marker. This particular combination of monoclonals to the two antigens was selected for our experiments because of the quite different patterns of reactivity of these antibodies with malignant tumours: Thy-1 mainly reacted with neuroblastoma and some soft tissue sarcomas while K20 mainly reacted with different carcinomas. No data on simultaneous usage of these markers in tumour phenotyping have been published as yet.

We have described here the distribution of two antigens identified by two Mabs, K20 and ICO-10, in a number of solid tumours. These antibodies belong to the group of Mabs specifically reacting with some tumour cells and are helpful for subvariant identification, staging and immunophenotyping of solid tumours.

K20 Mab specifically reacted with malignant cells of epithelial origin as well as with tumour cells in smooth muscles. This antibody usually identified endothelial cells, basal layer epithelium and a small subset of lymphocytes in tumour tissue sections. The tissue distribution of the antigen formed a broad network surrounding all the tumour cells in tissue sections. No reaction was noted in soft tissue sarcomas, neuroblastoma, neurosarcoma or malignant neurolemmoma.

Our experiments showed that ICO-10 Mab-detected Thy-1 antigen was expressed on cells of neuroblastoma, neurosarcoma, malignant neurolemmoma, teratoblastoma, rhabdomyosarcoma and some other soft tissue sarcomas. No antigen expression was found on tumour cells of epithelial origin. ICO-10 Mabs are helpful for diagnosing neuroblastoma metastases to lymph nodes, adrenal cortex and bone marrow. Immunodiagnosis of bone marrow neuroblastoma metastases using ICO-10 Mabs is of particular clinical interest (in normal bone marrow Thy-1 antigen expression is found only in 0.1% cells) since the difficulties arising in the course of morphological identification of metastatic neuroblastoma cells are well known.

Thus, the two types of antibodies used in our experiments, K20 (VLA-4) and ICO-10 (Thy-1) seem to be helpful for immunophenotyping of solid tumours when used in combination. These antibodies may be used as additional tools in the studies of human solid tumours alongside specific markers for malignant tumours, and the combination of the two antibodies seems helpful. The functional role of the antigens identified by these antibodies in tumour sections requires further studies.

The authors are grateful to Mrs O. Andrushkevish for general management and the translation of this article.

### References

AMIOT, M., BERNARD, A., THAN, H.C. & 2 others (1986). The human cell surface glycoprotein complex (gp120/200) recognized by monoclonal antibody K20 is a component binding to phytohemagglutinin on T-cells. *Scand. J. Immunol.*, **23**, 109.

BARYSHNIKOV, A.J. (1984). Monoclonal antibodies against human thymocyte differentiation antigens. *Bull. Exp. Biol.*, **101**, 324.

BARYSHNIKOV, A.J., MAKHONOVA, L.A., TUPITSYN, N.N. & 2 others (1985*a*). Immunodiagnosis of childhood T-cell hemoblastoses. *Sovetskaya Med.*, **N 11**, 33.

BARYSHNIKOV, A.J., TUPITSYN, N.N., KRYZHANOV, M.A. & 3 others (1985*b*). The panel of monoclonal antibodies and antisera for human hemoblastosis diagnosis. *Doklady AN SSSR*, **212**, 753.

BOURNE, B.A. (1983). *Handbook of Immunoperoxidase Staining Methods*. Dako Corporation: USA.

CASPER, J.T., BORELLA, L. & SEN, L. (1977). Reactivity of human brain antiserum with neuroblastoma cells and non-reactivity with thymocytes and lymphoblasts. *Cancer Res.*, **37**, 1750.

DANON, Y.L., SEEGER, R.C. & MAIDMAN, J.E. (1980). Fetal neural antigens on human neuroblastoma cells. *J. Immunol.*, **124**, 2925.

GROUX, H., HUET, S., VALENTINE, H. & 2 others (1989). Suppressor effects and cyclic AMP accumulation by the CD29 molecule of CD4$^+$ lymphocytes. *Nature*, **339**, 152.

JANOSSY, G. (1981). Membrane markers in leukaemia. In *The Leukaemic Cell*, Catovsky, D. (ed.) p. 129. Livingstone: Edinburgh.

McKENZIE, J.L. & FARBE, J.W. (1981). Human Thy-1: unusual localization and possible functional significance in lymphoid tissues. *J. Immunol.*, **126**, 843.

PETERSON, I.S., BARYSHNIKOV, A.J., TUPITSYN, N.N. & 3 others (1986). Morphological and cytochemical characteristics of childhood lymphosarcoma in case of various immunological subtypes. *Exp. Oncol.*, **8**, 45.

RETTING, N.G., DRACOPOLIN, N.C., CHESA, R.G. & 4 others (1985). Role of human chromosome 11 in determining surface antigenic phenotype of normal and malignant cells. *J. Exp. Med.*, **162**, 1603.

RITTER, C., SAUVAGE, C. & DELIA, D.H. (1983). Human Thy-1 antigen: cell surface expression on early T- and B-lymphocytes. *Immunology*, **49**, 555.

SEEGER, R.C., DANON, Y.L., RAYNER, S.A. & HOOVER, F. (1982). Definition of a Thy-1 determinant on human neuroblastoma, glioma, sarcoma and teratoma cells with monoclonal antibody. *J. Immunol.*, **128**, 983.

TUPITSYN, N.N., MECHETNER, E.B., BARYSHNIKOV, A.J. & 2 others (1987). Diagnosis of human leukemia erythroid types with the use of monoclonal antibodies. *Exp. Oncol.*, **9**, 30.

ZIKIRYAKHODZHAEV, D.Z., TUPITSYN, N.N., BARYSHNIKOV, A.J. & 1 other (1987). Studies of neuroblastoma with the use of a monoclonal antibody complex. *Exp. Oncol.*, **9**, 62.